# FSHD1 Diagnosis in a Russian Population Using a qPCR-Based Approach

**DOI:** 10.3390/diagnostics11060982

**Published:** 2021-05-28

**Authors:** Nikolay Vladimirovich Zernov, Anna Alekseevna Guskova, Mikhail Yurevich Skoblov

**Affiliations:** Research Centre for Medical Genetics, Laboratory of Functional Genomics, 1, Moskvorechie Str., 115478 Moscow, Russia; gousskova@gmail.com (A.A.G.); mskoblov@gmail.com (M.Y.S.)

**Keywords:** FSHD1 diagnosis, D4Z4 array, qPCR, genotyping

## Abstract

Facioscapulohumeral dystrophy (FSHD) is an autosomal dominant myodystrophy. Approximately 95% of cases of FSHD are caused by partial deletion of the D4Z4 macrosatellite tandem repeats on chromosome 4q35. The existing FSHD1 diagnostic methods are laborious and not widely used. Here, we present a comprehensive analysis of the currently used diagnostic methods (Southern blotting and molecular combing) against a new qPCR-based approach for FSHD1 diagnosis. We observed 93% concordance between the results obtained by the new qPCR-based approach, reference Southern blotting and molecular combing methods. Applying the qPCR-based approach in the studied population, we observed a prevalence (64.9%) of the permissive alleles in the range of 3–6 D4Z4 units for a group of patients, while in a group of carriers, the permissive alleles were mostly (84.6%) present in the range of 6–9 D4Z4 units. No prevalence of disease penetrance depending on gender was observed. The results confirmed the earlier established inverse correlation between permissive allele size and disease severity, disease penetrance. The results suggest the applicability of the qPCR-based approach for FSHD1 diagnosis and its robustness in a basic molecular genetics laboratory. To our knowledge, this is the first study of FSHD1 permissive allele distribution in a Russian population.

## 1. Introduction

Facioscapulohumeral dystrophy (FSHD) is a progressive skeletal muscle dystrophy with a prevalence from 1:20,000 to 1:8500 in observed populations, making it the third most common muscle dystrophy after Duchenne muscular dystrophy and myotonic dystrophy. FSHD is uniquely linked with subtelomeric macrosatellite tandem D4Z4 repeats of chromosome 4q35. In healthy individuals, the number of D4Z4 repeats varies from 11 to 150 [1,2]. A highly homologous D4Z4 repeats can be found on chromosome 10, but this repeats does not show linkage to FSHD [3]. Each D4Z4 repeat on chromosome 4 consists of about 3300 base pairs [4] and encodes the protein-coding retrogene DUX4 [5]. Expression experiments on FSHD myoblasts strongly suggested DUX4 pathogenic influence [6,7,8,9].

For chromosomes 4q35 and 10q26, two major haplotypes distal to D4Z4 array are described, the so-called A and B haplotypes [10,11]. The significant difference between the A and B is that only the A haplotype of chromosome 4 (4qA) contains the polyadenylation signal sequence for DUX4 mRNA and is associated with the expression of the DUX4 protein in skeletal muscles [8].

Two types of FSHD have been described: FSHD1 (MIM: 158900) and FSHD2 (MIM: 158901). FSHD1 is characterized by autosomal dominant inheritance and is caused by partial contraction of a 4qA-type D4Z4 array up to 1–10 repeats. More than 95% of all FSHD are FSHD1 cases [4,11,12,13]. FSHD2 accounts for the remaining 5% of cases. FSHD2 is caused by mutations in genes related to chromatin methylation status, such as SMCHD1, DNMT3B and LRIF1, where SMCHD1 can be found in more than 85% of cases [14,15].

Despite the genetic causes being different, both types of FSHD show relaxation of the 4q35 D4Z4 array chromatin in cis with the 4qA haplotype and activation of the ectopic expression of DUX4 from the most distal D4Z4 repeat [16].

Summarizing the aspects of molecular–genetic confirmation of FSHD1, the following complications can be assumed: requirement of genomic DNA of a high-molecular weight; discrimination between chromosome 4q35 and 10q26 D4Z4 repeats; determination of the exact number of D4Z4 repeats; determination of the A/B haplotype of the chromosome 4q35-D4Z4 repeat.

As FSHD1 accounts for more than 95% of cases, we focused on comprehensive analysis of methods for FSHD1 molecular diagnostics, such as Southern blotting and molecular combing [17,18,19] versus a newly developed qPCR-based method. The qPCR-based FSHD1 diagnostic test allows avoiding the laborious step of hybridization used in Southern blotting and molecular combing, making FSHD1 diagnostic available for a broad spectrum of diagnostic laboratories. To our knowledge, this is the first genotype study representing permissive allele distribution of FSHD1 patients and their unaffected relatives from Russia.

## 2. Materials and Methods

### 2.1. DNA Samples

Genomic DNA samples were obtained from patients with clinical signs of FSHD (*n* = 61) and their phenotypically healthy relatives (*n* = 40). Genetic constructs “C85” (6 D4Z4 units from chromosome 10q26, 10qA haplotype) and “λ260201” (3 D4Z4 units from chromosome 4q35, 4qA haplotype) were kindly provided by Dr. R. Lemmers (Leiden University Medical Center, Leiden, The Netherlands).

Genomic DNA was isolated from PBMC in agarose plugs as described previously [17]. The isolation of PBMC was performed by a standard Ficoll density gradient. Isolated PBMCs were used to generate high quality genomic DNA in agarose plugs as described previously [17], with the expectation that one agarose block contained about 7 µg of DNA (approximately 1 × 10^6^ cells).

### 2.2. The D4Z4 Array Analysis by Blotting

All the steps of the Southern blotting were performed as previously described [17]. Briefly, a set of four half-agarose plugs for each sample was prepared. Half of the agarose plugs were treated either by *Eco*RI/*Hind*III, *Eco*RI/*Avr*II (*Bln*I), *Xap*I or *Hind*III (Thermo Scientific, Waltham, MA, USA) in an appropriate restriction buffer (Thermo Scientific, Waltham, MA, USA). *Eco*RI/*Hind*III was used for sizing all alleles of the 4th and 10th chromosomes, *Eco*RI/*Avr*II was used for the 4th chromosome alleles’ sizing (10th chromosomes alleles eliminated by *Avr*II), *Xap*I was used for the 10th chromosome alleles’ sizing (4th chromosomes alleles eliminated by *Xap*I), and *Hind*III was used for haplotyping. For the determination of the D4Z4 arrays’ sizes and their homogeneity, the probes p13E-11 (D4F104S1) [20] and D4Z4 [20], respectively, were used. Probes A and B [11] was used for the determination of the haplotype of the D4Z4 arrays.

### 2.3. The D4Z4 Array Analysis by Molecular Combing

Molecular combing diagnostics by the Genomic Vision FSHD test (Genomic Vision, Bagneux, France) were performed according to the manufacturer’s protocol with the use of the “CombHeliX^®^ FSHD probes” (Genomic Vision, Bagneux, France).

### 2.4. qPCR-Based D4Z4 Arrays Length Estimation

#### 2.4.1. Pulsed-Field Gel Electrophoresis

Steps from the equilibration agarose blocks for treatment with endonuclease were performed as described previously [17].

The half-agarose blocks were treated by 30 U of *Eco*RI (Thermo Scientific, Waltham, MA, USA). After overnight digestion, the agarose plugs were used for pulsed-field gel electrophoresis (PFGE).

The following conditions for PFGE were used: 0.5× TB buffer (44.5 mM Tris base, 44.5 mM boric acid) supplemented with 10 mM DTT; 0.8% agarose gel (“Agarose, universal, peqGOLD” VWR Chemicals, Vienna, Austria); hexagonal electrode and electrophoresis chamber “Gene Navigator” (Pharmacia Biotech, Uppsala, Sweden); power supply unit “2301 MacroDrive 1 Power Supply” (LKB Bromma, Stockholm, Sweden); control unit “2015 Pulsaphor Plus Control Unit” (LKB Bromma, Stockholm, Sweden); electrophoresis chamber connected with “2219–001 Multitemp II Thermostatic Circulator” (LKB Bromma, Stockholm, Sweden). PFGE conditions were as follows: 2.5 L of 0.5× TB buffer with 10 mM DTT; voltage 450 V; 0.5 sec. of pulse; 3.5 h of total duration; buffer temperature +5 °C. We used two molecular weight markers: the Lambda Mix Marker, 19 (Thermo Scientific, Waltham, MA, USA) and the M12 DNA Ladder (SibEnzyme, Novosibirsk, Russia) in separate wells on both sides of the gel.

After PFGE, the agarose gel was stained in 300 mL of the 0.5× TB buffer with ethidium bromide of a 0.5 µg/mL final concentration. Then the gel was washed in 300 mL of distilled H_2_O 20 min × 3 times. The gel picture was taken by “ChemiDOC XRS+ system” (Bio-Rad Laboratories, Veenendaal, The Netherlands), and the scheme of gel fragmentation was made by the “Image Lab” software (Bio-Rad Laboratories, Veenendaal, The Netherlands). The number of gel fragments may vary depending on the required resolution of the D4Z4 array size. Furthermore, the samples’ gel lines were fragmented according to the scheme, and each gel fragment was placed into a separate 1.5 mL marked microcentrifuge tube. A gel fragment from the area outside of the samples lines was used as a control of the gel contamination by DNA (“empty gel”) in the electrophoresis chamber. After that, each gel fragment was melted at 95 °C for 5 min and diluted 10 times by PCR-grade water. The obtained solutions were melted at 95 °C for 5 min and used directly for the PCR and qPCR, without any DNA purification steps.

#### 2.4.2. Primers Design

The reference sequences of chromosome 4q35 (GeneBank: AF117653.3) and chromosome 10q26 (GeneBank: AY028079.1) were aligned by the Clustal Omega tool (https://www.ebi.ac.uk/Tools/msa/clustalo, accessed on 25 March 2021). Based on the alignment results, the chromosome 4 unique loci were selected for primers design by the Oligo 7 software (version 7.60, Vondelpark, Colorado Springs, CO, USA).

#### 2.4.3. PCR for Sequencing

PCR was performed on “DNA Engine Dyad” (Bio-Rad, The Netherlands) with 1.25 U of “Smar^NG^Taq” DNA-polymerase (Dialat Ltd., Moscow, Russia); 1× “Ampli” PCR-buffer (Dialat Ltd., Moscow, Russia); 1.2 M Betaine (Tokyo Chemical Industry Co., Ltd., Tokyo, Japan); 2.5 mM MgCl_2_ solution (Dialat Ltd., Moscow, Russia); and dNTPs (0.2 mMdATP, 0.2 mMdTTP, 0.2 mMdCTP, and 0.2 mMdGTP; Dialat Ltd., Moscow, Russia). The primers were as follows: 0.4 mM Rep1F 5′- TCCCACCCTCAGGCTCCTC-3′; 0.4 mM Rep1R1seq 5′-AGTGCAGACCAGGGCGCCG-3′. The PCR conditions consisted of an initial “hot-start” at 95 °C for 3 min, followed by 40 cycles: 95 °C—30 s, 62 °C—5 s, and 72 °C—15 s. The PCR product was 351 bp for chromosome 4q35 and 345 bp for chromosome 10q26.

Sanger sequencing of PCR-products was performed by the “DNA-diagnostic laboratory” (Moscow, Russia) on ABI PRISM 310 Genetic Analyzer (Applied Biosystems, Waltham, MA, USA). The obtained sequences were analyzed with FinchTV v.1.5 software.

#### 2.4.4. qPCR-System

qPCR was performed on the “StepOnePlus Real-Time PCR System” (Applied Biosystems, Waltham, MA, USA) in a final volume of 25 µL with 1.25 U of “Smar^NG^Taq” DNA-polymerase (Dialat Ltd., Moscow, Russia); 1× “Ampli” PCR-buffer (Dialat Ltd., Moscow, Russia); 1.2 M Betaine (Tokyo Chemical Industry Co., Ltd., Tokyo, Japan); 2.5 mM MgCl_2_ solution (Dialat Ltd., Moscow, Russia); dNTP (0.2 mM dATP, 0.2 mM dTTP, 0.2 mM dCTP, 0.2 mM dGTP; Dialat Ltd., Moscow, Russia). Primers were as follows: 0.4 mM of Rep1,5F 5′-GTGCTTGCGCCACCCACGT-3′; 0.4 mM of Rep1R 5′-GCCGCGCGGAGGCGGAG-3′; TaqMan zond: 1 mM zond2Rep1 5′ FAM-AGTCCGTGGTGGGGCTGGGG-BHQ1 3′. A total of 10 µL of each of the ten-times diluted gel fragments after PFGE was used as templates. A total of 10 µL of the ten-times diluted “empty gel” fragment was used for estimation of PFGE gel contamination by DNA during the PFGE steps. For the positive controls, the following templates were used: 8 µL of the ten-times diluted “empty gel” fragment was used with an addition of 100 ng (2 µL) gDNA; 8 µL of PCR-grade water with an addition of 100 ng (2 µL) gDNA. A total of 10 µL of PCR-grade water used as a no-template control. The PCR conditions consisted of an initial “hot-start” at 95 °C for 3 min; followed by 40 cycles: 95 °C—30 s, 62 °C—5 s, 72 °C—15 s. The expected size of the PCR product was 146 bp.

qPCR was performed in two replicates for each gel fragment. qPCR data were processed by the 2^−ΔΔCt^ method [21].

The difference between the two types of gel fragments was calculated: fragments outside the zone of the lines and fragments of the gel lines. An internal control was an agarose gel from an off-line area as it reflected the contamination of the agarose gel with targeted molecules due to the PFGE, staining and washing steps. Thus, changes in the type of samples outside of the zone of the gel lines were reduced to zero. Thus, the equation for calculating the relative number of target molecules in the sample was reduced to the form: N(i) = 2 ^− (Ct(i) − Ct(g))^, where N(i)—the relative concentration of target template in the studied fragment of the gel; Ct (g)—the average value of the threshold cycle in the sample from an off-line area (the control gel “G-”); Ct (i) was the average value of the threshold cycle in the studied fragment of the gel line.

Gel fragments with maximum relative concentrations of 4q35 D4Z4 repeats contained the targeted alleles. The number of repeats in the targeted allele was estimated by matching the gel fragments with their molecular weight marker on the gel fragmentation scheme.

### 2.5. PCR-Haplotyping

For haplotyping of the contracted (<11 D4Z4 units) alleles, we used the “tri-primer PCR” system [22] with several modifications of the PCR components: as a PCR template, 10 μL of the PFGE gel fragment that contained the contracted 4q35 D4Z4 array was used, in addition to 1.25 U of “Smar^NG^Taq” DNA-polymerase (Dialat Ltd., Moscow, Russia); 1× “Ampli” PCR-buffer (Dialat Ltd., Moscow, Russia); 2.5 mM MgCl_2_ solution (Dialat Ltd., Moscow, Russia); dNTP (0.2 mMdATP, 0.2 mMdTTP, 0.2 mMdCTP, 0.2 mMdGTP; Dialat Ltd., Moscow, Russia); 2.5% DMSO; 0.5 μM of the pLAM-FW primer (TCTGTGCCCTTGTTCTTCCGT); 0.25 μM of the pLAM-RV primer (CTGATCACCGAAGTTATGTAAACCAA); 0.5 μM of the AS-pLAM primer (CACAGGGAGGGGGCATTTTA). The cycling conditions were as follows: initial denaturation at 95 °C for 2 min, followed by 38 cycles of 95 °C for 15 s, 60 °C for 15 s, and 72 °C for 15 s and final extension at 72 °C for 2 min. As a control of 4qA and 10qA haplotypes, 10pg per reaction of the λ260201 and C85 cosmids [4], respectively, were used. Whenever the 4qA allele is present in a sample, it will result in the presence of two bands (242 bp and 147 bp), whereas the presence of the 10qA allele will result in amplification of only one band (242 bp).

### 2.6. Statistical Analysis

A Chi-square test of independence was performed to examine the relation between gender and the disease penetrance. The Mann–Whitney U test was performed to analyze the significance of differences in the D4Z4 array length distribution in the group of patients and carriers. For all tests, differences were considered statistically significant for a *p*-value of ≤0.05. Statistical analyses were performed using online software (https://www.socscistatistics.com, accessed on 26 May 2021).

## 3. Results

### 3.1. qPCR System for 4q35 D4Z4 Array Length Estimation

**Figure 1:**
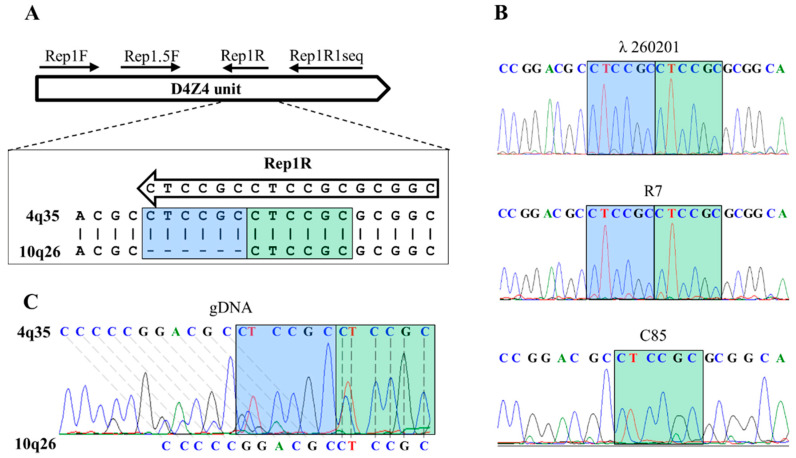
Differences in D4Z4 sequences of chromosomes 4q35 and 10q26. (**A**) Schematic representation of primers’ positions within D4Z4 unit. The position of each primer indicated by a black arrow and signed on top. The alignment of regions of the six-nucleotide differences is presented below the primers scheme. Two CTCCGC repeats are unique for chromosome 4q35 and are highlighted by two colored rectangles. The 4q35-specific primer Rep1R sequence is placed inside a black arrow. In the case of PCR for sequencing, the primers pair Rep1F + Rep1R1seq was used; in the case of qPCR for 4q35 D4Z4 arrays sizing, the primers pair Rep1.5F + Rep1R was used. (**B**) Sequence of PCR products obtained by using Rep1F + Re1R1seq primers pair and control matrices: λ260201 (4q35 specific cosmid); R7 (PFGE gel fragment with 4q35 D4Z4 array of DNA sample with known sizes of D4Z4 arrays); C85 (10q26 specific cosmid). In colored rectangles CTCCGC repeats are highlighted. (**C**) Sequence of PCR products obtained by Rep1F + Re1R1seq primers pair and native gDNA matrix. The sequence contains two types of the D4Z4 repeats: 4q35-specific (top row) and 10q26-specific (bottom row).

The alignment of chromosomes 4 and 10-derived D4Z4 sequences revealed a 1% sequence variation, of which one is a six-nucleotide difference (Figure 1A); for chromosome 4, there were two copies of a CTCCGC repeat, whereas chromosome 10’s D4Z4 reference sequences had only one copy of this repeat. The six-nucleotide difference was used to establish a chromosome 4q35-specific qPCR system. Specificity of the qPCR system is achieved by annealing the 3′-end of the reverse primer to the CTCCGC repeat. Unique amplification of a chromosome 4 DNA template (λ260201), but not of a chromosome 10 DNA template (C85), demonstrated the specificity of the qPCR system (Figure 1B). We validated this analysis on five DNA samples and identified in each sample the sequences of two different D4Z4 repeats, either with one or with two copies of the CTCCGC repeats, belonging to chromosome 10 or 4, respectively (Figure 1B, C). Subsequently, we treated the gDNA sample with a known permissive allele size (7 D4Z4 units) by EcoRI and performed PFGE. The gel fragment corresponding to the length of the permissive allele was excised and used as a template for PCR with the Rep1F + Rep1R1seq primer pair. The presence of two CTCCGC repeats in the sequence clearly suggests its 4q35 origin (Figure 1B).

**Figure 2:**
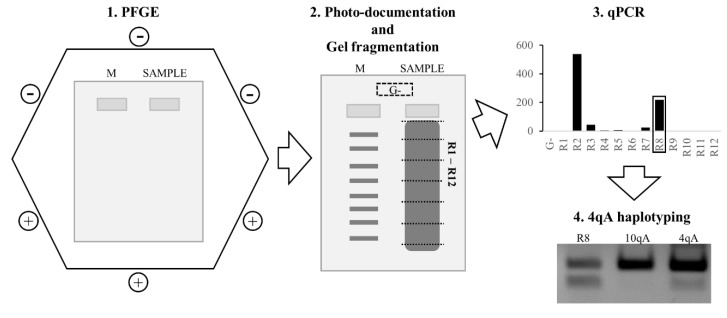
Workflow scheme of the qPCR-based FSHD1 diagnostic approach. Firstly, an agarose-embedded gDNA sample is digested by endonuclease and resolved by PFGE. Secondly, the PFGE–agarose gel is stained in ethidium bromide and photographed. The gDNA lane fragmentation scheme is marked on a photo. The agarose gel lane with digested gDNA is manually fragmented by scalpel according to the fragmentation scheme; usually, 12 gel fragments are obtained. Additionally, for the estimation of agarose gel contamination by the target DNA, the “G-” gel fragment is sliced from the off-line area. Thirdly, all the fragments used as a template for chromosome 4 D4Z4 repeat-specific qPCR. Comparing the qPCR data with the gel fragmentation scheme (position of the gel fragments with maximum target gel concentration against molecular weight marker), a conclusion regarding the length of the D4Z4 array is made. Fourthly, based on the qPCR data, the gel fragment with the maximum concentration of target DNA (R8) is used for 4qA haplotyping by allele-specific PCR. λ260201 and C85 are used as a control for the 4qA and 10qA haplotypes, respectively. Thus, the described approach provides information about the length of the chromosome 4 D4Z4 arrays and their haplotype—the minimum contributions for an FSHD1 diagnostic approach.

In order to avoid hybridization during the steps used in the blotting and in the molecular combing, the following approach for chromosome 4 D4Z4 array length estimation was used: agarose-embedded DNA preceded treatment by EcoRI (no AvrII was needed because the qPCR system is specific for 4q35 D4Z4 repeats) endonuclease; the obtained restriction fragments were separated by PFGE in a 0.8% agarose gel; the gel was photo-documented, and the gel fragmentation scheme was marked on a photo; the agarose gel line was manually fragmented, strictly according to the gel fragmentation scheme; the obtained gel fragments were used as a template for the 4q35-specific D4Z4 qPCR system. Conclusions regarding the length of the 4q35 D4Z4 arrays were made in two steps: first—based on the qPCR, we found the gel fragments with the largest quantity of 4q35 D4Z4 DNA; second—collating the position of these gel fragments against the molecular weight marker on the gel fragmentation scheme allowed us to define the 4q35 D4Z4 arrays sizes. For the PCR haplotyping, the gel fragments containing the contracted 4q35 D4Z4 arrays were used as a template. The resulting algorithm of the qPCR-based FSHD1 diagnostic approach is presented in Figure 2.

### 3.2. Comparison of the qPCR-Based 4q35 D4Z4 Array Length Estimation against Blotting and Molecular Combing Results

First, 46 individuals were analyzed by reference methods: by the Southern blotting (27 patients, 13 unaffected relatives); by the molecular combing (2 patients); by both methods (3 patients) (Supplementary Table S1).

**Figure 3:**
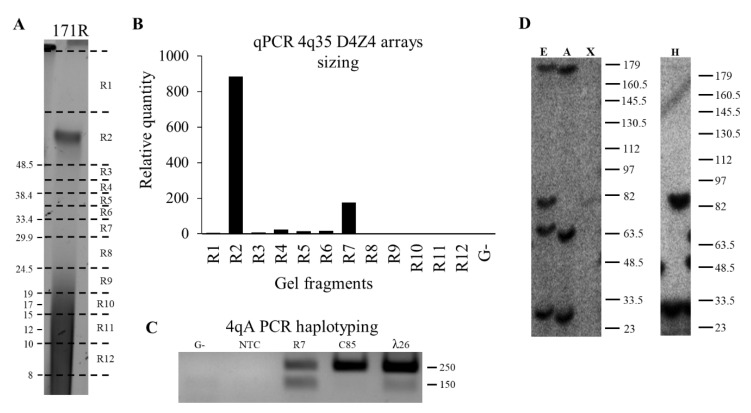
Results of the FSHD1 diagnostic test for patients. (**A**) PFGE results of the DNA sample of patient 171. Gel line 171R—*Eco*RI digested sample. The line 171R was manually fragmented; the gel fragment borders are indicated by a horizontal dotted black line. The gel fragment order is indicated on the right. The molecular weight marker scale is indicated on the left. (**B**) qPCR data were obtained using the gel fragments as a template. “G-” is negative gel control from the out-off sample’s line zone; R1–R12 correspond to gel fragments from R1 to R12. Based on the qPCR results, patient 171 had two 4q35 D4Z4 arrays in gel fragments R2 (normal size array, >48.5 kb) and R7 (contracted array up to 7 D4Z4 repeats). (**C**) PCR haplotyping results. NTC—negative control without agarose gel; G-—control of agarose gel contamination using G- gel fragment as a PCR template; R7—result obtained using the gel fragment R7 as a PCR template; C85—result obtained using 10 pg of cosmid C85 as a PCR template, control sample of the 10qA haplotype; λ26—result obtained using 10 pg of cosmid λ260201 as a PCR template, control sample of the 4qA haplotype. (**D**) Southern blotting results of patient 171’s DNA sample. On the left—results of hybridization with the p13e11 probe for D4Z4 arrays sizing and chromosome affiliation. A—*Eco*RI digested sample. E—*Eco*RI/*Avr*II digested sample. X—*Xap*I digested sample. On the right—results of hybridization with the A probe for D4Z4 arrays haplotyping. H—*Hind*III digested sample. The molecular weight marker scale is indicated on the right of each blot image.

After that, we validated the qPCR-based approach on the blotting and molecular combing reference results. The available reference DNA samples of 29 patients (at least one contracted allele, *n* = 20; no contracted allele, *n* = 9) and 13 healthy relatives (one contracted allele, *n* = 5; no contracted allele, *n* = 8) were used for the qPCR-based diagnostic test. In 39 (93%) of the cases, the qPCR-based results of the D4Z4 arrays length estimation were concordant with the reference. Among them, 20 individuals had one contracted type A allele; on the qPCR plot data, such type of samples have one major peak in gel fragments above the 48 kb threshold and one peak in gel fragments below the 48 kb threshold (Figure 3). One individual had two contracted type A alleles; both peaks were in gel fragments below the 48 kb threshold. One individual has two contracted alleles, but only one was the 4qA type; both peaks were in gel fragments below the 48 kb threshold (supplementary Figure S1). Additionally, 17 individuals had no contraction, and on qPCR plot data, only one peak in gel fragments was above the 48 kb threshold (Supplementary Figure S2).

For one individual (2%, one contracted allele), we failed to estimate the D4Z4 length, presumably because of low amount of DNA available or its low quality, and for two unrelated individuals the results were false negatives (5% of all cases, both had one contracted type A allele). For the false negative DNA samples, we repeated PFGE, sliced out gel fragments with the expected localization of the contracted 4q35 alleles and used them as a template in the PCR system for sequencing (Re1F + Rep1R1seq primers). As this PCR-system is a nonspecific system for any type of the D4Z4 arrays, we expected that amplification should not be affected by sequence issues. However, we observed no amplification, and the most relevant explanation for such a result was a mix-up with the samples. Unfortunately, the patients refused a complete reanalysis, and we referred to these samples as discordant ones.

In the studied cohort of phenotypically FSHD patients, we observed 10 individuals with no FSHD1 permissive alleles. Approximately 5% of all the FSHD cases are FSHD2 type; one of the main features of FSHD2 is the hypomethylation of the D4Z4 arrays <25%, whereas healthy individuals have >35% [23,24]. To measure the methylation status of the D4Z4 arrays in these patients, methylation-sensitive Southern blotting [15] was kindly performed by Dr. van der Vliet (LUMC, The Netherlands); the results are presented in Supplementary Table S1. Among the analyzed cases, one patient (136) had clear FSHD2 signs, with D4Z4 arrays hypomethylation down to 2%; one patient (153) had border line methylation status (31%); for five patients (104, 124, 147, 148, 150), methylation was in a healthy range, and both types of FSHD were excluded for them; for three individuals (115, 126, 128), diagnosis was not complete due to uninterpretable or no results of the methylation assay.

### 3.3. Comparison of 4qA PCR-Haplotyping Blotting and Molecular Combing Results

For the 27 patients and 13 unaffected individuals, we performed 4qA haplotyping by PCR haplotyping [22]. The group was the same as for qPCR analysis, with the exception of the two discordant individuals. According to the reference results, among them were 17 individuals having no contracted 4q35 D4Z4 alleles, but at least one of the alleles with the A haplotype; two individuals with two contracted alleles; and 21 individuals with one contracted type A allele.

As a PCR template, we used the gel fragments containing chromosome 4-derived D4Z4 arrays. It should be mentioned that we did not repeat the PFGE procedure, but the gel fragments obtained previously were utilized for the qPCR-based diagnostic test. Concerning the individuals who had no contracted 4q35 D4Z4 arrays, we used the gel fragments directly above the 48 kb border as a PCR template. These gel fragments contained a mix of D4Z4 alleles (normal sized D4Z4 arrays from chromosomes 4 and 10) if the reference sample contained at least one non-contracted 4qA allele; we expected the same allele based on PCR haplotyping. The results of the sequencing of the PCR products of the reference chromosome 4 (λ260201) control, untreated gDNA samples and the PCR product of gel fragment amplification clearly demonstrated the specific amplification of the 4q35 A haplotype on the gel fragments template (Supplementary Figure S3).

From the 39 individuals analyzed, we observed completely concordant results of the haplotyping. It is significant that, in the case of a patient with two contracted 4q35 D4Z4 arrays, only the allele of the 4qA type was amplified (Supplementary Figure S1, gel fragment R10). For the one individual for whom the qPCR-based approach failed to estimate the 4q35 D4Z4 array length, we also failed to perform PCR haplotyping.

### 3.4. Analysis of the Permissive Alleles Distribution in the Russian Population

Additionally, we performed D4Z4 length estimation by a qPCR-based approach and 4qA PCR haplotyping for 28 patients and 27 unaffected relatives, who had not been diagnosed either by blotting or molecular combing.

**Figure 4:**
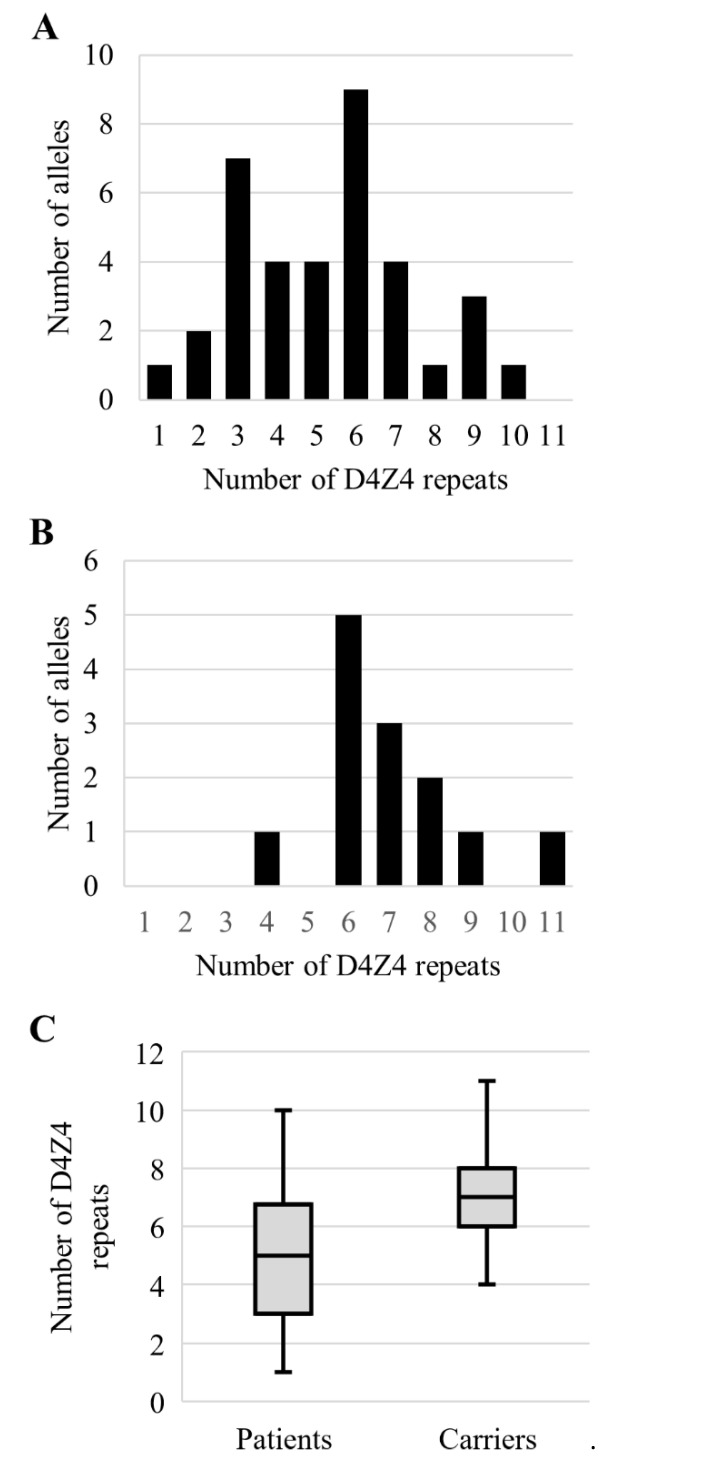
D4Z4 array size distributions. Permissive alleles size distribution among affected individuals (**A**), phenotypically healthy relatives (**B**). (**C**) Comparison of the alleles distribution in groups of patients and phenotypically healthy carriers (z-score = −2.56, *p* = 0.01).

Summarizing the results of the FSHD1 diagnostic test by blotting, molecular combing and qPCR-based 4q35 D4Z4 length estimation followed by 4qA PCR haplotyping in a group of 61 affected individuals, FSHD1 was confirmed for 37 (60.7%) patients. Among the confirmed cases were 24 (64.9%) individuals having permissive alleles with 3–6 D4Z4 units. In group of 40 healthy relatives, we diagnosed 13 (32.5%) carriers of the permissive 4qA allele. Amongst carriers, 11 (84.62%) had the permissive 4qA allele with the number of D4Z4 units from 6 to 9 (Figure 4). The carriers had statistically longer permissive alleles (z-score = −2.56, *p* = 0.01). We did not observe disease penetrance prevalence being dependent on gender (χ2 = 1.29, *p* = 0.26).

## 4. Discussion

Recently, high-throughput NGS approaches have been widely used for diagnosis of inherited disorders and have actively displaced other methods. However, some objects are difficult or impossible to study by the NGS, especially macrosatellite tandem repeats. An increasing number of studies of macrosatellites have proved their functional significance in different cellular processes [25,26]. Thus, it is necessary to have methods for detecting changes in macrosatellite tandems. For FSHD1, the blotting diagnostic test is still the most frequently used, with well-known advantages, such as a relatively high throughput, a wide range of chemistry on the market, the possibility of several rehybridizations of one blot and its long-time storage. However, not all equipment is easily available, especially in the case of Southern blotting, either radioactive or nonradioactive. Additionally, there are FSHD1 cases with results difficult to interpret due to the presence of hybrid alleles, mosaicism and D4Z4 array rearrangements. Increasingly used, the molecular combing approach has resolved some of the Southern blotting limitations and provided information from a single experiment on the length of the D4Z4 arrays on chromosomes 4 and 10, including their A/B haplotypes, mosaicism level and structure of the complicated alleles. Currently, the number of samples per experiment is limited to four, and another limitation is due to the special equipment and chemistry needed.

The minimum contribution for an FSHD1 diagnostic test is the presence of a contracted 4q35 D4Z4 array ranging from 1 to 10 D4Z4 units in linkage with the so-called A haplotype. Blotting and molecular combing are quite laborious and require the use of special equipment; additionally, not all the information is used for diagnosis. In addition, recently, the haplotyping of an SSLP sequence proximal to the D4Z4 array was proposed as a refining criterion for the FSHD diagnostic test. Currently, there are controversial results regarding the predictive value of SSLP haplotyping [27,28], and studies in different populations are needed to clarify which of the SSLP haplotypes are linked with FSHD1 in specific populations.

To simplify the diagnostic test, focusing only on 4q35 D4Z4 array sizing and its A haplotyping, we developed a qPCR-based approach followed by PCR-A haplotyping for an FSHD1 diagnostic test. For this, a chromosome-specific PCR was developed based on a six-nucleotide difference between chromosomes 4 and 10.

It should be mentioned that the rare non-permissive chromosome 10 haplotype 10A176T [16] has the two six-nucleotide repeats, as well as AvrII restriction sites identical to chromosome 4. Another rare chromosome 4 haplotype, 4A166, is currently considered as non-permissive. In these haplotypes, the D4Z4 arrays contractions detected either by the Southern blotting or a qPCR-based approach will look similar to permissive 4q35 haplotypes. Without additional SSLP sizing, both approaches are unable to differentiate the 10A176T and 4A166 from the 4q35 permissive haplotypes. However, these are relatively low in frequency, with up to 2.5% of the 10A176T and up to 4.1% of the 4A166 haplotypes occurring in studied populations; correctly collected familial medical history can minimize the chances of the misdiagnosis caused by the 10A176T or 4A166 alleles.

During the first step of the comprehensive analysis of the blotting and molecular combing against the qPCR-based approach, we focused on 4q35 D4Z4 array sizing in a group of 29 FSHD1 patients and 13 healthy individuals. The vast majority of the comparison results coincided (93%) in the range of +/- 2 D4Z4 units. This range could be explained by the following reasons: in the case of Southern blotting, the definition of the allele size depends on a visual comparison of the allele signal with the molecular weight marker, which is quite subjective; the PFGE conditions are also varied across the presented methods; in the case of the qPCR-based approach, manual gel line fragmentation could also influence the accuracy of the allele sizing.

The presence of one controversial result is explained by an insufficient quality or quantity of DNA. To avoid issues linked to the DNA quality, it is necessary to use fresh blood for PBMC isolation, as well as using DNase free reagents and performing complete deproteinization. During the gel fragmentation, minimization of UV exposure could also preserve DNA quality. As for the Southern blotting procedure, the optimum DNA input for the qPCR-based D4Z4 array sizing is about 3 micrograms; such an amount of DNA allows one to clearly resolve specific signals and background noise.

Comparison of the D4Z4 sizing between the reference methods and the qPCR-based diagnostic test revealed two discordant cases. We tried to understand if these events were caused by the inability of the qPCR system to amplify the target alleles in these DNA samples. Thus, we repeated the PFGE and sliced out the gel fragments, which should have contained contracted 4q35 D4Z4 alleles, and used them as a template in the PCR system for sequencing (Re1F + Rep1R1seq primers). It is important that this PCR system is not a specific system for any type of the D4Z4 arrays. However, we observed amplification only in controls, thus pointing to the absence of any D4Z4 arrays in the examined ranges of length for both cases. The most likely explanation of the mismatches is that the samples were simply mixed up.

Overall, the obtained data allow us to conclude that the qPCR approach successfully determined the length of the D4Z4 array of chromosome 4 along with standard diagnostics. Here, for the qPCR-based approach for D4Z4 sizing, we used a molecular weight marker with fragments from 8 to 48 kb; if the studied sample had no 4q35 D4Z4 arrays less than 48 kb, the exact number of the D4Z4 units could not be estimated. In this case, the only conclusion that could be made is the absence of a 4q35 D4Z4 contracted allele. However, if necessary, a molecular weight marker with a bigger size of fragments and longer time of PFGE could be used.

Although the developed approach does not currently provide information about the A/B haplotype of contracted alleles, the PCR assays for A/B haplotyping published earlier [22,29] could be used to detect the target haplotype in PFGE gel slices of interest. Potentially, a multiplex qPCR system with primers for D4Z4 repeats and for the 4qA haplotype could be used to accelerate FSHD1 diagnosis.

To our knowledge, this is the first study of permissive alleles in a Russian population. It was shown for other populations that permissive alleles are mostly between 4 and 8 D4Z4 units [28,30]. Similar results were observed in our study, with most presented permissive alleles in the range of 3–6 D4Z4 units. In unaffected carriers, the alleles’ size is mostly between 6 and 8 D4Z4 units. This observation confirms the phenomenon of inverse correlation between permissive allele size and disease severity, penetrance and age of onset [31]. In addition, in presented study, the carriers of the permissive alleles are first-degree relatives of the FSHD patients; their unaffected state could be explained either by additional genetic and/or epigenetic components, by gender or by age at the time of study. To clarify the exact mechanism, further clinical studies are needed.

Interestingly, FSHD commonly does not have an impact on life expectancy; however, for the individuals with 1–3 D4Z4 repeats in the permissive alleles, the situation may differ. As shown earlier, infantile FSHD is usually caused by permissive alleles with 1–3 D4Z4 repeats and characterized by severe features and fast progression [32,33,34]. Thus, the lower amount of the patients with 1–3 D4Z4 repeats on a permissive allele could be explained by shortened life expectancy. In the case of patients with 8–10 D4Z4 repeats on a permissive allele, the lower number of cases could be explained by low penetrance or minimal FSHD features, which may be unnoticed by many patients for a long time.

## Data Availability

The datasets used and/or analyzed during the current study are available from the corresponding author on reasonable request.

## Supplementary Materials

The following are available online at https://www.mdpi.com/article/10.3390/diagnostics11060982/s1, Figure S1: Results of FSHD diagnostic for patient with two contracted 4q35 D4Z4 arrays. Figure S2: Results of FSHD diagnostic for healthy individual. Figure S3: The sequences flanking the DUX4 polyadenylation signal within the pLAM region. Table S1: Results of FSHD1 diagnostic by Southern blotting, qPCR-based approach and molecular combing.
