# Supplementary material for: FSHD1 Diagnosis in a Russian Population Using a qPCR-Based Approach
Source: Diagnostics (Basel). 2021 May 28;11(6):982. doi: 10.3390/diagnostics11060982 (PMC8226754; doi:10.3390/diagnostics11060982)
Supplement: Supplementary file 1 [file diagnostics-11-00982-s001.zip › diagnostics-1182000 Supplementary 5.28.pdf]

Supplementary

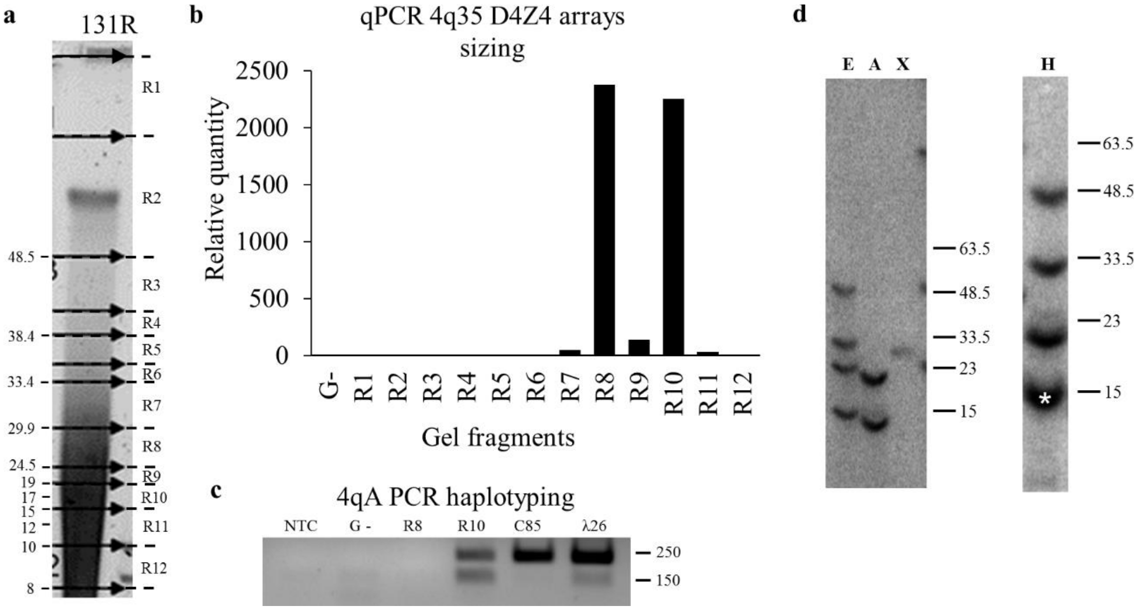

Supplementary Figure S1. Results of FSHD diagnostic for patient with two contracted 4q35 D4Z4 arrays. A – PFGE results of patient's №131 DNA sample. Gel line 131R – *Eco*RI digested sample. The gel line 131R manually fragmented, the gel fragments borders indicated by horizontal black arrows. The gel fragments' order indicated on the right. The molecular weight markers scale indicated on the left. B – qPCR data obtained using the gel fragments as a template. "G-" is negative gel control from the out off sample's line zone; R1-R12 corresponds to gel fragments from R1 to R12. Base on qPCR results patient №131 has two 4q35 D4Z4 arrays in gel fragments R8 and R10, thus having alleles with 6 and 3 D4Z4 repeats. C – PCR-haplotyping results, NTC – negative control without agarose gel; G- – control of agarose gel contamination using G- gel fragment as a PCR template; C85 – result obtained using 10 pg of cosmid C85 as a PCR template, control sample of the 10qA haplotype; λ26 – result obtained using 10 pg of cosmid λ260201 as a PCR template, control sample of the 4qA haplotype. R8 – result obtained using the gel fragment R8 as a PCR template, absence of the PCR products indicate that 6 repeats D4Z4 array does not belong to 4qA haplotype; R10 – result obtained using the gel fragment R10 as a PCR template, presence of two PCR products (as in λ26) indicate that 3 repeats D4Z4 array belongs to 4qA haplotype. D – Southern blotting results of patient's №131 DNA sample; on the left – results of hybridization with the p13e11 probe for D4Z4 arrays sizing and chromosome affiliation. E – *Eco*RI digested sample. A – *Eco*RI/*Avr*II digested sample. X – *Xap*I digested sample. On the right – results of hybridization with the A probe for D4Z4 arrays haplotyping. H – *Hind*III digested sample. The molecular weight markers scale indicated on left of each blot image. White asterisk – nonspecific hybridization signal.

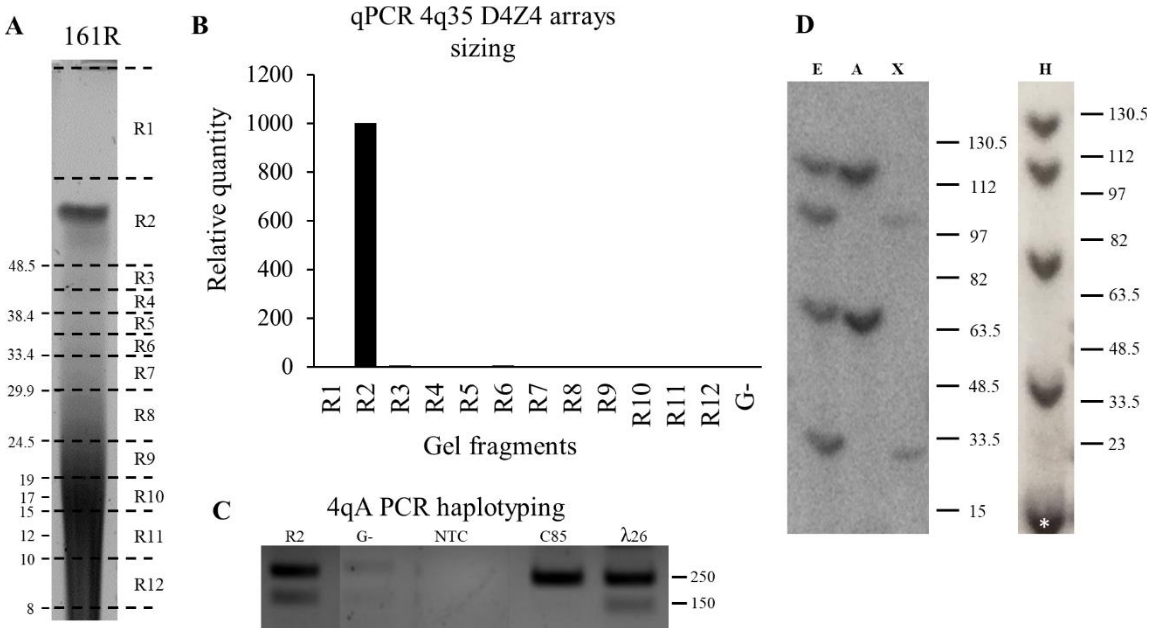

Supplementary Figure S2. Results of FSHD diagnostic for healthy individual. A – PFGE results of individual's №161 DNA sample. Gel line 161R - *Eco*RI digested sample. The designations like in the supplementary figure 1. B – qPCR data, designations like in the supplementary figure 1. Base on qPCR results individual №161 has no 4q35 D4Z4 arrays less than 48.5 kb. C – PCR-haplotyping results, designations like in the supplementary figure 1. R2 – result obtained using the gel fragment R2 as a PCR template, presence of two PCR products (as in λ26) indicate that the D4Z4 array belongs to 4qA haplotype. D – Southern blotting results of individual's №161 DNA sample, designation like in the supplementary figure 1. White asterisk – nonspecific hybridization signal.

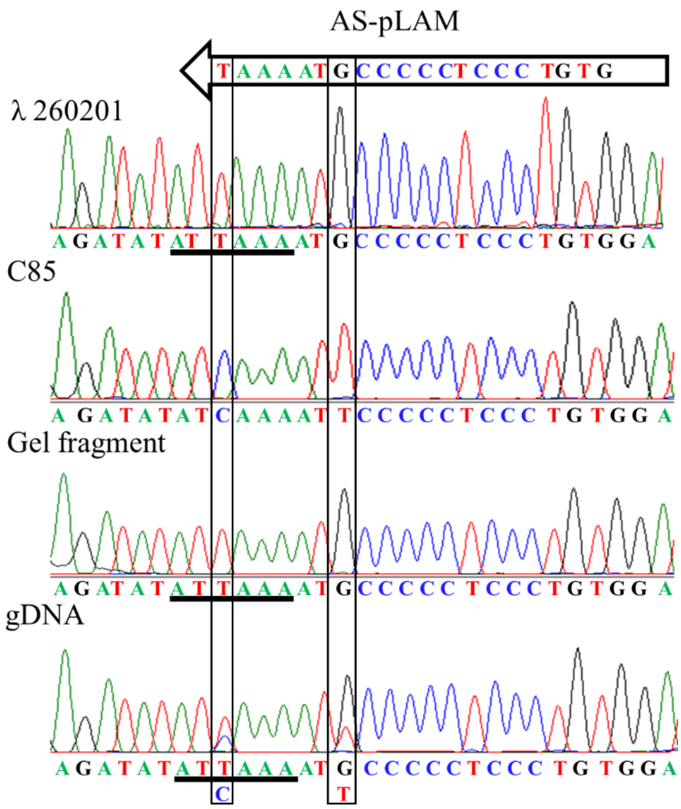

Supplementary Figure S3. The sequences flanking the DUX4 polyadenylation signal within the pLAM region. For PCR the pLAM-FW and pLAM-RV primers were used [1]. The polyadenylation sequence (ATTAA) of the DUX4 is underlined by black horizontal lines. Sequence of the allele specific primer AS-pLAM is highlighted in black arrow. The control templates presented by λ 260201 (4qA specific) and C85 (10qA specific). The Gel fragment is a template obtained from gel slice with pathogenic sized 4q35 D4Z4 array, the sequence clearly demonstrate presence of the 4qA specific sequence in the gel slice. The gDNA template is a sequence of not digested gDNA, the sequence demonstrate presence of both – 4qA specific and 10qA specific D4Z4 arrays in the template.

Supplementary Table S1. Results of FSHD1 diagnostic by Southern blotting, qPCR-based approach and molecular combing. RU - number of D4Z4 repeat units; U - the result is not interpretable; P - FSHD1 permissive allele; NP – FSHD1 non-permissive allele. Discordant cases highlighted in red. Controversial case highlighted in yellow. Alleles with > 11 RU are non-permissive for FSHD1.

ID	Family ID	Affected / not affected	Sex	Southern blotting						qPCR						Molecular combing						Methylation status (%)
				Allele1			Allele2			Allele1			Allele2			Allele1			Allele2			
				RU	haplotype	status	RU	haplotype	status	RU	haplotype	status	RU	haplotype	status	RU	haplotype	status	RU	haplotype	status	
32	-	a	F	5	A	P	21	B	NP	7	A	P	>11	-	NP	-	-	-	-	-	-	-
104	-	a	F	28	B	NP	47	A	NP	>11	A	NP	>11	-	NP	-	-	-	-	-	-	48
115	-	a	F	13	B	NP	23	A	NP	>11	A	NP	>11	-	NP	-	-	-	-	-	-	U
120	-	a	M	6	A	P	53	A	NP	7	A	P	>11	-	NP	-	-	-	-	-	-	-
123	-	a	F	3	A	P	53	A	NP	>11	-	NP	>11	-	NP	-	-	-	-	-	-	-
124	-	a	M	29	U	NP	47	U	NP	>11	A	NP	>11	-	NP	-	-	-	-	-	-	53
125	-	a	M	U	U	U	U	U	U	>11	-	NP	>11	-	NP	3	A	P	26	B	NP	6
126	-	a	F	U	U	U	U	U	U	>11	A	NP	>11	-	NP	51	A	NP	35	B	NP	-
128	-	a	M	32	A	NP	47	A	NP	>11	A	NP	>11	-	NP	-	-	-	-	-	-	U
130	Fam1	a	M	6	A	P	32	A	NP	6	A	P	>11	-	NP	-	-	-	-	-	-	-
137		n	M	26	A	NP	32	A	NP	>11	A	NP	>11	-	NP	-	-	-	-	-	-	-
131	Fam2	a	F	3	A	P	6	B	NP	3	A	P	6	not A	NP	-	-	-	-	-	-	-
132		n	F	6	B	NP	47	A	NP	7	A	NP	>11	-	NP	-	-	-	-	-	-	-
133	-	a	F	4	A	P	42	U	NP	U	U	U	U	U	U	-	-	-	-	-	-	-
134	-	a	F	7	A	P	9	A	P	8	A	P	10	A	P	9	A	P	9	A	P	-
135	-	a	F	3	A	P	30	A	NP	4	A	P	>11	-	NP	-	-	-	-	-	-	-
136	-	a	M	24	A	NP	47	A	NP	>11	A	NP	>11	-	NP	27	A	NP	50	A	NP	2
139	-	a	F	5	A	P	38	A	NP	4	A	P	>11	-	NP	-	-	-	-	-	-	-
143	Fam3	a	M	8	A	P	U	U	U	9	A	P	>11	-	NP	-	-	-	-	-	-	-
144		n	M	8	A	P	18	U	NP	8	A	P	>11	-	NP	-	-	-	-	-	-	-
146	-	a	M	3	A	P	24	U	NP	3	A	P	>11	-	NP	3	A	P	25	A	NP	-
147	-	a	F	20	U	NP	28	U	NP	>11	A	NP	>11	-	NP	-	-	-	-	-	-	53
148	-	a	F	42	A	NP	42	A	NP	>11	A	NP	>11	-	NP	-	-	-	-	-	-	54
149	-	a	M	2	A	P	57	B	NP	4	A	P	>11	-	NP	-	-	-	-	-	-	-
150	Fam4	a	M	19	A	NP	20	A	NP	>11	A	NP	>11	-	NP	-	-	-	-	-	-	52
151		n	F	18	A	NP	19	A	NP	>11	A	NP	>11	-	NP	-	-	-	-	-	-	41
152	-	a	F	4	A	P	20	B	NP	4	A	P	>11	-	NP	-	-	-	-	-	-	29
153	-	a	F	13	A	NP	34	A	NP	>11	A	NP	>11	-	NP	-	-	-	-	-	-	31
154	-	a	M	6	A	P	14	B	NP	6	A	P	>11	-	NP	-	-	-	-	-	-	-
155	Fam5	a	M	6	A	P	23	A	NP	7	A	P	>11	-	NP	-	-	-	-	-	-	-
156		n	F	6	A	P	34	A	NP	7	A	P	>11	-	NP	-	-	-	-	-	-	-
161	-	n	M	19	A	NP	35	A	NP	>11	A	NP	>11	-	NP	-	-	-	-	-	-	-
162	Fam6	a	M	3	A	P	13	A	NP	3	A	P	>11	-	NP	-	-	-	-	-	-	-
163		n	F	13	A	NP	62	A	NP	>11	A	NP	>11	-	NP	-	-	-	-	-	-	-
165	-	a	F	5	A	P	U	U	U	3	A	P	>11	-	NP	-	-	-	-	-	-	-
166	Fam7	n	M	26	A	NP	59	A	NP	>11	A	NP	>11	-	NP	-	-	-	-	-	-	-
167		a	M	3	A	P	23	B	NP	3	A	P	>11	-	NP	-	-	-	-	-	-	-
168		n	M	23	B	NP	26	A	NP	>11	A	NP	>11	-	NP	-	-	-	-	-	-	-
169		n	F	46	A	NP	49	A	NP	>11	A	NP	>11	-	NP	-	-	-	-	-	-	-
170	Fam8	n	F	7	A	P	11	A	NP	7	A	P	>11	-	NP	-	-	-	-	-	-	41
171		n	F	7	A	P	18	B	NP	7	A	P	>11	-	NP	-	-	-	-	-	-	27
108		a	M	7	A	P	26	B	NP	7	A	P	>11	-	NP	-	-	-	-	-	-	34

1. Papanikos F, Skoulatou C, Sakellariou P, Kekou K, Christopoulos TK, Kanavakis E, Traeger-Synodinos J, Ioannou PC: **A simplified approach for FSHD molecular testing**. *Clin Chim Acta* 2014, **429**:96-103.
